# Investigation of Materials for Enhanced Vibrational Insulation in Ground Structures Using Concrete Mixtures: A Case Study of Rubber Aggregate Addition

**DOI:** 10.3390/ma17133092

**Published:** 2024-06-24

**Authors:** Maciej Gruszczyński, Alicja Kowalska-Koczwara, Tadeusz Tatara

**Affiliations:** Faculty of Civil Engineering, Cracow University of Technology, 31-155 Kraków, Poland; maciej.gruszczynski@pk.edu.pl (M.G.); tadeusz.tatara@pk.edu.pl (T.T.)

**Keywords:** rubber concrete, concrete design, vibrational insulation, ground barriers

## Abstract

The reduction of vibrations in concrete has been a topic of discussion among scientists. This article presents research on designing concrete mixes for constructing ground barriers with enhanced vibration isolation using waste materials. This study discusses the design of concrete mixes for the construction of concrete partitions with increased vibration isolation, using the polish standards. The experiments were conducted at the Laboratory of Building Materials Engineering at the Cracow University of Technology as part of the project entitled “Innovative construction of vibration-insulating barriers to protect the environment from transport vibrations and similar sources”. The concrete composition utilized blast furnace cement CEM III/A 42.5 N, with mineral and chemical additives. Recycled rubber aggregate from used tires was employed to enhance vibration isolation. Measurement results demonstrated the effectiveness of the concrete in dampening vibrations, confirming its suitability for practical use.

## 1. Introduction

Buildings in busy urban areas are constantly affected by various sources of mechanical vibrations. One of the main causes is the transportation infrastructure, including the constant movement of vehicles on roads [1,2], the rhythmic sounds of tramway systems [3,4], and the steady rumble of trains on railways [5]. These urban activities generate continuous mechanical vibrations that spread through the surrounding environment and affect nearby structures. Paraseismic vibrations [6], have an essential impact on affected buildings despite being invisible to the naked eye.

The way a building responds to these dynamic forces goes beyond simple physics; it is a complicated process influenced by the building’s inherent dynamic response characteristics [7]. It is not just about the strength or frequency of the outside disturbance, but rather a complex interaction of structural dynamics where the building’s properties determine its ability to withstand vibrations and subsequent behavior.

In structural engineering, understanding dynamic characteristics is crucial for designing buildings that can withstand city life and keep people safe and comfortable. Engineers analyze and employ creative designs to provide buildings with a strong balance, enabling them to endure city vibrations and ensure a comfortable environment. Dynamic response characteristics include natural vibration frequencies, modes of vibration, and vibration damping, which determine how a building interacts with and responds to external forces [8].

The natural vibration frequencies of a structure represent its intrinsic vibrational patterns, similar to the resonant frequencies of a musical instrument. These frequencies correspond to specific modes of vibration and can be elucidated using computational methods like the finite element method (FEM).

In the case of dynamic response, vibration damping is a crucial factor as it controls how energy is dissipated within a structure. Unlike natural frequencies and modes, damping cannot be accurately determined through computational methods alone. Instead, it requires physical measurements to accurately assess its damping characteristics. This is because damping is influenced by various factors such as material properties, structural geometry, and environmental conditions.

In structural engineering, damping is essential for reducing vibrations, preventing structural fatigue, and ensuring the accuracy of structural analyses. The critical damping ratio (D) is used to describe damping properties [9]. However, the logarithmic decrement (denoted as δ) provides a more comprehensive understanding of damping by measuring the gradual decrease in vibration amplitude over successive cycles.

The characterization of damping properties is crucial in structural dynamics, where empirical observation and theoretical abstraction converge to understand structural behavior. Engineers are constantly seeking optimized damping strategies for resilient and responsive structures.

Three main methods can be used to protect buildings and their occupants from vibrations: addressing the source of the vibrations, mitigating vibrations as they travel, and implementing measures within the building. Addressing the source is best performed during the construction or upgrading of the transportation infrastructure, such as installing anti-vibration systems in rail networks. Mitigating vibrations along their path is less common due to cost and space limitations, but it can be important for reducing road vibrations. Lastly, isolating vibrations within the building can be achieved through various methods, from anti-vibration mats to specialized structural elements. However, these solutions, especially advanced measures like vibration dampers, can be expensive.

As part of the POIR.04.01.02-00-0001/17 project, new vibration-isolating barriers were developed, which can also be used as excavation linings or walls of underground parking lots. One innovation is a concrete mix containing rubber granules that help reduce vibrations in structures such as palisades. A rubber granulate from used tires with a fraction of 2/6 mm was selected. The chosen rubber granulate is produced through mechanical crushing in dry conditions at ambient temperature, which guarantees the appropriate stability and repeatability of the chemical composition and grain size. This project aims to improve the resilience of buildings in urban areas exposed to vibration disturbances.

There is some research, in which the use of rubber aggregate was considered [10,11]. There are also review articles that summarize and compare independent research studies, evaluating whether these aggregates can potentially improve the behavior of concrete elements [12,13,14].

## 2. Concrete Design Assumptions

The foundational principles guiding the formulation of concrete mixtures aimed at crafting concrete barriers endowed with superior vibration isolation capabilities were meticulously crafted in alignment with the stringent directives laid out in the industry standards. These standards, specifically [15], titled “Concrete—Requirements, properties, production, and conformity”, and [16], dubbed “Execution of special geotechnical works—Bored piles”, serve as the bedrock upon which the design assumptions were firmly established. Drawing upon the comprehensive guidelines delineated within these standards, the project embarked on a journey of meticulous planning and strategic formulation, ensuring that every facet of the concrete mixture would adhere to the highest industry benchmarks. From the initial stages of conceptualization to the final execution, the project team rigorously adhered to the specifications outlined in these standards, leveraging their wealth of knowledge and expertise to navigate the complexities inherent in the construction of vibration-resistant barriers. These standards provided a comprehensive framework encompassing a myriad of considerations, ranging from the fundamental requirements of concrete composition to the intricacies of execution in specialized geotechnical contexts. By anchoring the design assumptions in these well-established standards, the project fostered a culture of excellence and precision, laying the groundwork for the creation of resilient and enduring concrete barriers capable of withstanding the rigors of dynamic urban environments.

Preliminary assumptions were made regarding the basic technical properties of the concrete mixture and hardened concrete for the construction of palisades, including the following:

- Compressive strength class: minimum C30/37;
- Exposure class XA1 (i.e., concrete exposed to contact with the natural ground and groundwater), water/cement ratio ≤ 0.55;
- Maximum aggregate grain size: Dmax = 16 mm;
- Consistency class S4/S5 (i.e., Abram’s cone slump ≥ 200 mm), maintained for at least 60 min;
- Water penetration depth under pressure according to [17] not exceeding 45 mm.

Additionally, in the process of designing concrete mixtures, additional requirements for concrete intended for the execution of bored and displacement piles formed in the ground were taken into account, as outlined in Annex D of the [15] standard (see Table D.1), including the following:

- Minimum cement content Cmin ≥ 325 kg/m^3^;
- Content of fine fractions (i.e., particles ≤ 0.125 mm including cement and additives) ≥ 400 kg/m^3^.

The selection of materials for the concrete mixtures was a meticulous process aimed at ensuring optimal performance and the longevity of the constructed barriers. Commonly available gravel aggregates were meticulously chosen for their compatibility and accessibility, providing a sturdy foundation for the mixtures. For the binder, blast furnace cement CEM III/A 42.5 N sourced from “Cementownia Nowiny” emerged as the prime candidate. Its exceptional strength characteristics, coupled with high cement paste density, limited shrinkage, and its favorable thermal properties instilled confidence in its ability to uphold the structural integrity of the geotechnical structures over time. To further enhance the workability and pumpability of the concrete mixtures, as well as to meet regulatory requirements regarding the content of fine fractions, a mineral additive of type II was incorporated into the design. Specifically, the silica fume fly ash category, compliant with the [18] standard, was chosen for its proven efficacy in enhancing concrete performance while maintaining the required standards of quality and durability. In addition to the mineral additive, the inclusion of EnveroMix 178 rheological admixture played a pivotal role in ensuring the consistency and longevity of the mixtures. Its compatibility with the selected cement and fly ash underscored its suitability for the project, ensuring consistent performance and stability for a minimum duration of 60 min, critical for construction operations. Furthermore, to augment the vibration isolation capabilities of the barriers, a strategic decision was made to incorporate rubber granulate sourced from used tires with a fraction size of 2/6 mm. Chosen for its resilience and damping properties, this additive mitigates the transmission of vibrations, thereby enhancing the effectiveness of constructed barriers in reducing urban vibrations and ensuring the comfort and safety of occupants.

**Figure 1:**
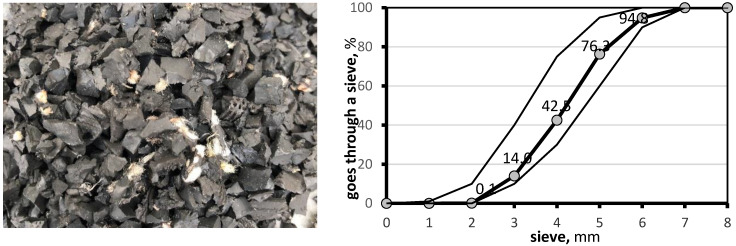
Grain size curve and view of the used rubber granulate 2/6 mm (Thin line—scatter, thick line—measurement).

The selected rubber granulate is produced through mechanical crushing in dry conditions at ambient temperature, ensuring appropriate stability and consistency in chemical composition and grain size. Figure 1 shows the grain size curve of the used rubber granulate 2/6 mm. 

The used rubber granulate of fraction 2/6 mm from used car tires is characterized by the following properties (manufacturer’s data):

- Bulk density 380–600 kg/m^3^;
- Volumetric density 1100–1250 kg/m^3^;
- Humidity ≤ 0.5%;
- Hardness IRHD—40–80 according to [19], method M;
- Ash content ≤ 18.5%;
- Content of washable zinc ≤ 0.5 mg/dm^3^;
- Content of metallic impurities ≤ 0.02%;
- Content of mineral impurities ≤ 0.02%.

## 3. The Results of Preliminary and Proper Research

The initial phase of research undertaken as part of the ongoing program was dedicated to formulating a recipe for foundational concrete, devoid of any vibration isolation modifiers. The primary objective was to achieve specific parameters outlined by the [15] standard, namely a compressive strength class of C30/37 XA1, a consistency class of S4/S5, a maximum aggregate grain size of 16 mm (Dmax 16), and a water-to-cement ratio (Cl) of 0.20. These parameters serve as crucial benchmarks in ensuring the structural integrity and performance of the concrete mixture in various environmental conditions.

**Figure 2:**
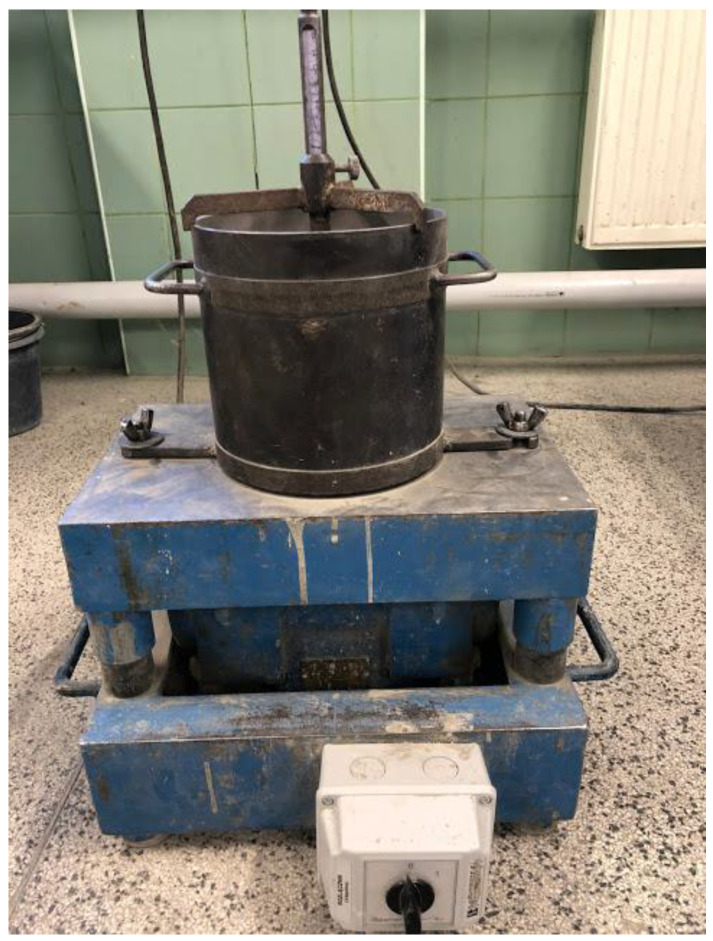
A cylinder with a volume of 5 dm^3^ mounted on a vibrating table for determining the bulk density of aggregate (iterative method of selecting a rubble stack with max. density).

As part of this project, sieve analyses of aggregates were carried out and the composition of the mineral mixture with the maximum density of the rubble stack was determined by the iterative method—successive approximations in a steel cylinder with a volume of 5 dm^3^ (Figure 2).

The result of the conducted iteration was the determination of optimal proportions, guaranteeing the maximum density of the rubble stack, for the weight mixing of aggregates from the Borzęcin mine, namely

*Sand 0/2:Gravel 2/8:Gravel 8/16 = 1:0.65:0.80.*

**Figure 3:**
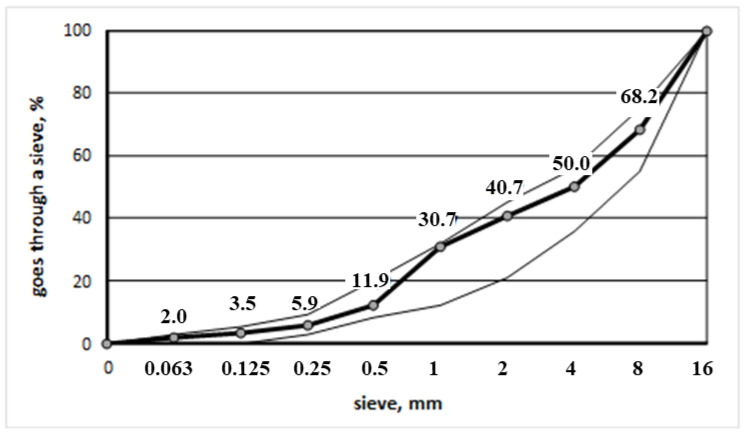
Grain size distribution curve of the Borzęcin aggregate mixture (Thin line—scatter, thick line—measurement).

In Figure 3, there is the grain size distribution curve of the subject aggregate mixture.

The recipe developed during this phase, delineated in Table 1, serves as the cornerstone for subsequent experimentation and refinement. It encapsulates the meticulous blend of materials and proportions necessary to meet the stringent requirements set forth by the standard, laying the groundwork for further optimization and customization tailored to the specific demands of the project.

The comprehensive analysis of both the properties of the concrete mixture and the characteristics of the hardened concrete substantiated the attainment of the targeted parameters following a 28-day curing period. These parameters encompassed various crucial aspects, including but not limited to the consistency class and its sustained stability over time, the compressive strength of the concrete, and the depth of water penetration under pressure. Through meticulous testing and evaluation, it was conclusively demonstrated that the concrete mixture not only met but exceeded the prescribed standards, thereby affirming its suitability for the intended application. The consistency class, indicative of the workability and flowability of the fresh concrete, remained consistent throughout the curing process, ensuring uniformity and reliability in construction practices. Moreover, the compressive strength of the hardened concrete met the designated requirements, attesting to its structural integrity and load-bearing capacity. This parameter serves as a fundamental indicator of the concrete’s ability to withstand external forces and stresses, thereby ensuring the longevity and durability of the constructed elements. Furthermore, the evaluation of water penetration depth under pressure provided valuable insights into the concrete’s resistance to moisture ingress, a critical factor in safeguarding against deterioration and damage due to environmental exposure. The confirmed achievement of these parameters underscores the efficacy of the concrete mixture in meeting performance expectations and fulfilling the requisite standards for quality and durability.

Given the aforementioned factors, a strategic decision was made to alter the composition of the standard concrete C35/45 by introducing rubber granulate. Specifically, varying proportions of rubber granulate were proposed, with 10, 15, 20, 25, and 30% of the 2/8 mm aggregate fraction being replaced accordingly. This deliberate adjustment aimed to explore the impact of rubber granulate on the properties of the concrete mixture, particularly in terms of vibration isolation and mechanical performance. To ensure uniformity and consistency in the mixtures, meticulous attention was paid to regulating their fluidity and workability. This was achieved through the precise dosing of the rheological admixture Chryso EnveroMix 178. By carefully adjusting the dosage of the admixture, the intention was to attain the desired consistency level, characterized by a cone slump measurement exceeding 200 mm. This approach was vital in maintaining the desired flow characteristics of the concrete while accommodating the incorporation of the rubber granulate modifier. In essence, this methodical approach to modifying the concrete mixture sought to strike a balance between incorporating innovative materials for enhanced performance and preserving the essential properties necessary for effective construction practices. Through systematic experimentation and meticulous control of mixture parameters, the aim was to optimize the blend to meet both performance requirements and practical application needs.

The industrial batches were meticulously crafted within the confines of a well-equipped precast concrete plant, known for its adherence to high standards of quality and precision. With meticulous attention to detail, each batch was carefully calibrated to contain precisely 3.5 m^3^ of concrete, a volume chosen to guarantee not only the consistency and homogeneity of the mixture but also to faithfully replicate the conditions encountered in large-scale industrial concrete production processes. Following the meticulous batching process, where every component was measured and combined with precision, the freshly prepared concrete mixture underwent the next phase of its journey. It was seamlessly transferred into the rotating drum of a specialized concrete mixer truck, a pivotal step that marked the transition from production to transportation. This transfer ensured the preservation of the mixture’s integrity while preparing it for the rigors of transit to its final destination. Once inside the drum, the concrete mixture underwent a thorough mixing process, meticulously orchestrated to last precisely 60 min. This duration, coupled with low-speed rotations, emulated the real-life conditions encountered during the transportation of concrete from the production facility to various construction sites. This careful blending not only facilitated the uniform distribution of components but also ensured the preservation of the mixture’s desired properties, essential for achieving optimal performance and durability in the final concrete structures.

From each batch, samples of concrete mixture were taken for the following tests:

- Determination of consistency using the slump cone method, according to the [20].
- Determination of concrete mixture density, according to the [21]
- Air content testing using the pressure method, according to the [22]

Additionally, cubic concrete samples with a side length of 150 mm were prepared from the obtained mixture. These samples were used for compressive strength testing at intervals of 7, 28, 56, and 90 days, and for determining the water penetration depth under pressure.

During the tests at the concrete plant, the consistency of the concrete mixture and its behavior over time (i.e., after 10 and 60 min) were analyzed, along with the air content in the compacted concrete. The dosage of the rheological admixture Chryso EnviroMix 178 was adjusted to ensure the desired mixture consistency (200–250 mm, i.e., S4/S5 according to the [15]).

Practical test results showed that depending on the recipe and the type of vibration isolation additive, the quantity of EnviroMix 178 admixture ranged from 2.40 to 2.75 kg/m^3^, which was 0.40–0.75 kg/m^3^ less than the amount established in laboratory conditions (i.e., 3.15 kg/m^3^).

Air content measured using the pressure method in the compacted concrete mixture ranged from 0.4 to 1.7%, meeting the project requirements.

A detailed summary of the results of the consistency testing of concrete mixtures and air content is presented in Table 2.

The analysis of the consistency of concrete mixtures and the examination of air content, meticulously detailed in Table 2, signifies a successful adherence to the predefined standards set forth by the project. These results not only meet but also align closely with the project’s stringent requirements, underscoring the reliability and accuracy of the experimental procedures employed. Of particular note is the consistent behavior observed in the concrete mixtures during the critical period spanning from 10 to 60 min, reflecting a robust stability and performance characteristic crucial for real-world applications. Furthermore, the findings from the industrial-scale testing exhibit a remarkable parallelism with those obtained under controlled laboratory conditions. This correlation underscores the validity and applicability of the laboratory-derived insights in practical settings, offering assurance regarding the reliability and consistency of the experimental outcomes. However, it is essential to highlight a nuanced aspect observed during the industrial testing phase: a reduction in the dosage level of the rheological additive Chryso EnviroMix 178. While this adjustment may seem minor, it warrants careful consideration as it could potentially impact the long-term performance and characteristics of the concrete mixtures. Therefore, further scrutiny and possibly iterative refinement of the dosage levels may be warranted to ensure optimal performance and durability in real-world applications.

In Table 3, the results of the concrete strength tests for the production of palisades after 7, 28, 56, and 90 days of curing are compiled. In accordance with the provisions of PN-B-06265:2018-10 [23], considering the use of blast furnace cement CEM III/A 42.5 N in concrete production, it was decided to consider the strength measured after 90 days as the decisive indicator. The tests were conducted following the PN-EN 12390-3 [24] procedure using the MATEST C070 strength-testing machine.

The findings gleaned from the tests provided the necessary insights to identify the most suitable concrete blend, which incorporates a 10% infusion of 2/6 mm rubber granulate, for further field trials. This meticulously chosen mixture promises to offer a balance of structural integrity, enhanced vibration isolation properties, and durability, making it well suited for practical applications in real-world scenarios.

To ensure the reliability of the concrete parameters acquired, a thorough examination was undertaken in accordance with the [17] protocol. This procedure meticulously assessed the maximum depth to which water could penetrate under pressure. Following the guidelines outlined in [23], the examination spanned a duration mirroring a 90-day timeframe, ensuring comprehensive analysis. The culmination of these efforts is reflected in the comprehensive data presented in Table 4, encapsulating the outcomes of this rigorous evaluation.

The findings derived from assessing the maximum water penetration depth, as per the [17] standard, across all concrete mixtures incorporating vibration isolation additives, reveal a reassuring outcome. In each case, the measured depths fall below the prescribed threshold of 45 mm, denoting a notable deviation from the maximum allowable limit. This substantial margin signifies a robust adherence to the specified requirements, offering a considerable buffer against potential water ingress under pressure.

**Figure 4:**
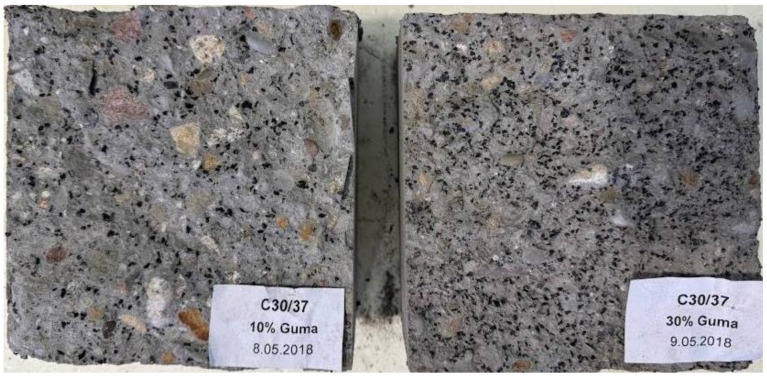
Cross-section of concrete samples for the construction of a barrier with vibration isolation additives in the form of 2/6 mm rubber granulate, with 10% (on the **left**) and 30% (on the **right**) content.

After testing the concrete sample with vibration isolation additives, it was cracked open, and the uniformity of the distribution of the additive within the concrete structure was evaluated (see Figure 4).

This thorough examination of the cross-sections of concrete samples, each containing vibration isolation additives, provided valuable insights. It showcased a consistent dispersion of these additives, evenly spread throughout the no clustering or accumulation was observed in any specific regions of the samples. This observation serves as compelling evidence affirming the design of these concretes. Despite their notably fluid nature, denoted by their S4/S5 consistency rating, they demonstrate a remarkable absence of segregation tendencies. Such uniform distribution underscores the precision engineering behind these concrete formulations, ensuring their structural integrity and efficacy in vibration isolation applications.

To make a final assessment of the suitability of the C30/37 concrete mixture with a 10% addition of rubber granulate for the construction of the palisade, a field trial was conducted on the construction site. As part of the trial, four piles with a diameter of Ø400 mm and a length of 6 m were produced from the C30/37 concrete mixture with a 10% addition of 2/6 mm rubber granulate, manufactured on-site under industrial conditions. During the field trial, the concrete mixture was carefully monitored for consistency and workability, ensuring that it met the required standards. Special attention was paid to the placement and compaction of the concrete around the reinforcement to ensure structural integrity and stability. The performance of the piles was evaluated based on their ability to withstand the load and environmental conditions typical of the construction site. This included assessing factors such as compressive strength, resistance to vibration, and durability over time. Additionally, the field trial provided valuable insights into the practical aspects of working with the concrete mixture on-site, including handling, pouring, and curing procedures. Any observations or challenges encountered during the trial were documented for further analysis and refinement of the concrete mixture formulation and construction techniques. Overall, the field trial served as a crucial step in validating the performance and practical feasibility of using the C30/37 concrete mixture with rubber granulate additives for constructing the palisade, providing valuable data for future projects and optimizations in concrete construction practices.

**Figure 5:**
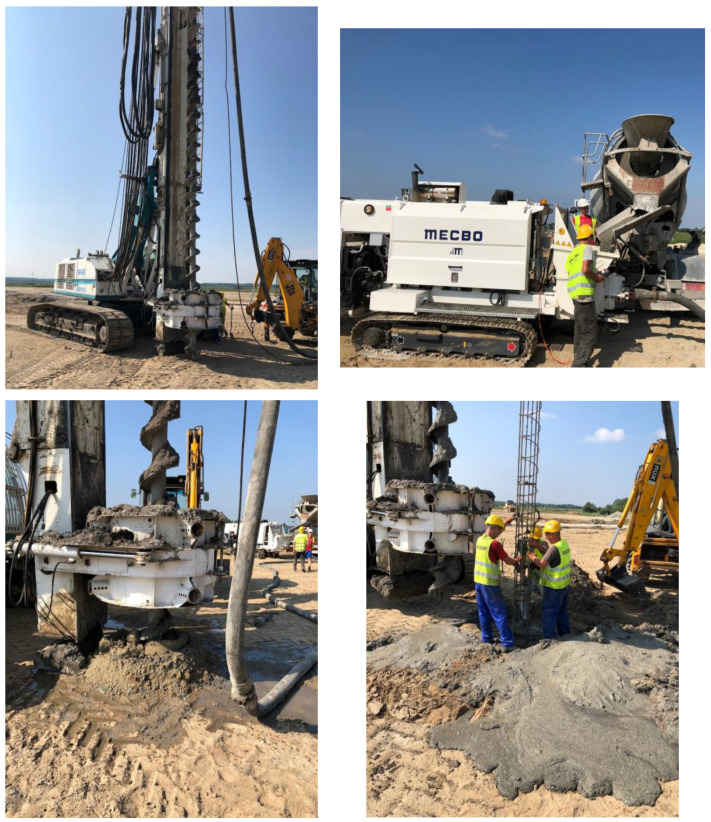

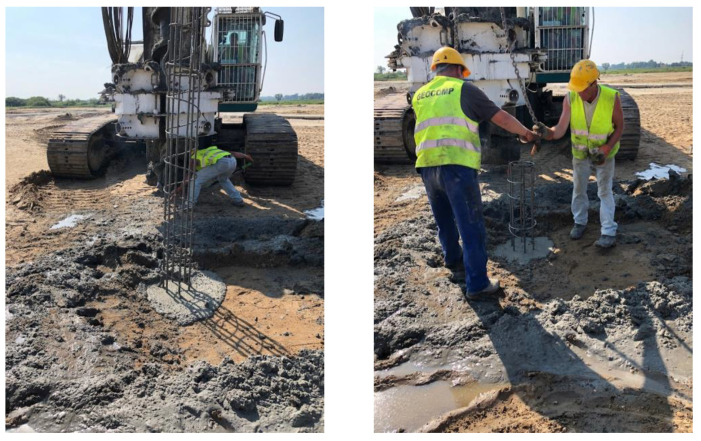
View from the implementation of the field trial for pile driving. The pile driver, the concrete pumping test, and the formed 400 mm diameter pile with the immersed reinforcement cage are visible.

At the same time, a practical evaluation of the rheological properties of the concrete mixture was conducted, along with a pumping test over a distance of 60 m and an assessment of the feasibility of immersion in the formed concrete column of a steel reinforcement cage (see Figure 5).

During the evaluation of the rheological properties of the concrete mixture, its flowability, workability, and consistency were closely monitored. The aim was to ensure that the mixture could be easily pumped over a considerable distance while maintaining its desired properties. The pumping test involved the use of concrete pumps to transport the mixture from the batching plant to the construction site over a distance of 60 m. This test provided valuable insights into the performance of the concrete mixture during transportation, including its ability to flow smoothly through the pump system without segregation or blockages. Simultaneously, the feasibility of immersion in the formed concrete column of a steel reinforcement cage was assessed. This involved lowering a preassembled steel cage into the freshly poured concrete column to evaluate its ability to penetrate the mixture without encountering significant resistance or affecting the concrete’s integrity. The practical evaluation under real-world conditions allowed for a comprehensive assessment of the concrete mixture’s behavior and performance during various construction activities. Any issues or challenges encountered during the tests were addressed promptly to ensure the successful implementation of the concrete mixture in the construction project.

During the field trial, the concrete producer assessed the basic properties of the concrete as part of the in-house production control. The results of the tests for the C30/37 class concrete samples with a 10% addition of 2/6 mm rubber granulate are presented in Table 5.

The properties of the concrete mixtures and hardened concrete obtained from laboratory and industrial tests met the adopted assumptions—both in laboratory-scale experiments and in conditions of production at the precast concrete plant.

The request from the contractor arose from the specific demands of the construction process, which required an extended period of workability for the concrete mixture. This was particularly crucial given the challenging conditions, including higher temperatures, which can accelerate the setting time of concrete. To address this requirement, the authoring team embarked on a thorough exploration of potential solutions.

After consulting with various suppliers specializing in chemical admixtures for concrete, CHRYSO Plast CER 50 emerged as the most promising candidate for addressing the contractor’s needs. This particular admixture is known for its dual functionality: it acts as both a plasticizer, enhancing the workability and flowability of the concrete, and a retarder, slowing down the setting process. This combination makes it an ideal choice for extending the workability time of the concrete mixture while maintaining optimal consistency.

To validate the effectiveness of CHRYSO Plast CER 50 in real-world conditions, industrial-scale batches of concrete mixtures were meticulously prepared, adhering to the specified proportions and incorporating 10% rubber granulate as per the project requirements. The dosage of CHRYSO Plast Cer 50 was carefully determined at 0.8% by mass of cement, ensuring that the desired properties of the concrete would be achieved without compromising its performance.

After preparing the mixture, it was placed in the concrete mixer and left rotating at low speed. Consistency testing was then conducted using the slump cone method (according to [24]) at intervals of 10, 60, 120, 180, 240, and 300 min. Detailed results of the consistency behavior of the respective mixtures are presented in Table 6.

The thorough examination of the concrete mixtures’ consistency for palisade construction revealed that the CHRYSO Plast CER 50 admixture blends seamlessly with the CEM III/A 42.5 N Nowiny cement, fly ash, and CHRYSO EnwiroMix178 rheological admixture, ensuring consistent performance for an extended duration of up to 5 h. This capability becomes particularly crucial in scenarios characterized by high ambient temperatures, such as during the summer months, where prolonged transport distances of the mixture and the fabrication of large-scale elements are common challenges.

Moreover, to assess the effects of prolonging the setting time on the properties of hardened concrete, specimens were crafted from both sets of mixtures after 300 min. These specimens took the form of 10 cubic bodies, each measuring 150 mm on all sides. Following the procedures outlined in [24], these samples were stored for a duration of 28 days. Subsequently, their compressive strength was evaluated. The resulting average strength value, fcm = 47.2 N/mm^2^, with a comfortable margin, ensures the attainment of the targeted concrete strength class C30/37 as per [15]. This alignment with the initial design assumptions underscores the viability of extending the working time of these mixtures for up to 5 h by incorporating the CHRYSO Plast CER 50 admixture.

## 4. Discussion

The discussion about reducing vibrations by enhancing the damping properties of concrete is an important issue that has been drawing the attention of scientists from various parts of the world for many years. The significance of this problem lies in its potential benefits, such as reducing seismic vibrations, compared to the costs of producing concrete with such properties. However, in the context of construction in prestigious locations, especially in historical centers of tourist cities, an additional aspect arises.

In these areas, where there is high demand for real estate and the price per square meter is significant, apartment buildings are designed with discerning clients in mind who seek exceptional comfort and prestige. These clients are willing to pay more for the luxury of living in the heart of the city while maintaining a high standard of living. Therefore, despite the potential challenges associated with producing specialized concrete with enhanced vibration damping properties, there is significant demand for such solutions in the construction industry, where not only functionality matters but also prestige and quality of life [27].

The concept of damping in construction, as highlighted in reference [28], is frequently misconstrued, and often narrowly associated with the application of vibration dampers. However, it is essential to recognize that the construction itself possesses inherent damping characteristics, which are influenced by various factors such as the materials employed, structural connections, and the surrounding environment. Essentially, the building functions as a sort of filter, interacting with external vibrations and mitigating their effects through its inherent damping properties. Understanding and harnessing these intrinsic damping qualities can significantly impact the structural performance and resilience of buildings in dynamic environments.

**Figure 6:**
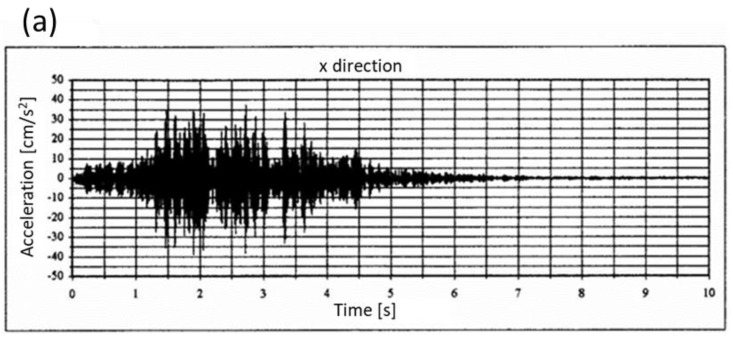

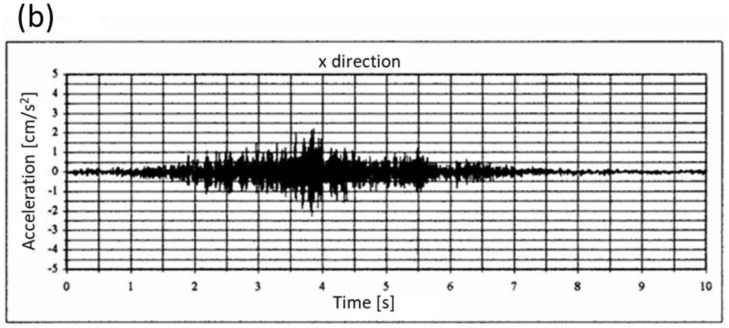
Vibration waveforms from passing metro trains: (**a**) vibrogram recorded on the ground just before the building, (**b**) vibrogram recorded at a rigid connection point of the building, at its foundation level.

In Figure 6, an exemplary vibration waveform was recorded on the ground just before the building, as well as the vibration waveform recorded on the foundation wall at a rigid connection point of the structure, are depicted, as per reference [29].

The significant reduction in vibrations from the ground to the building, as depicted in Figure 6, underscores the importance of enhancing the damping properties of materials. This reduction is significant, indicating a substantial decrease in vibrations upon entering the building structure. Given this notable effect, it becomes apparent that investing in improving the inherent damping characteristics of materials is a worthwhile endeavor. As cited in references [28,30], this approach offers a simpler and more cost-effective solution compared to the alternative of employing mechanical vibration dampers. By focusing on material enhancements, construction projects can achieve effective vibration reduction while optimizing cost-efficiency and simplifying implementation.

In [31], the performance of concrete mixtures incorporating 5%, 7.5%, and 10% of discarded tire rubber as both aggregate and cement replacements was thoroughly investigated. The study aimed to understand the impact of incorporating varying amounts of rubber on the overall characteristics and performance of the concrete. The results demonstrated that when up to 5% of the traditional concrete components were replaced with discarded tire rubber, there were no significant changes in the concrete’s key properties such as compressive strength, workability, and durability.

Two types of tire rubber, crumb rubber and tire chips, were used as fine and coarse aggregates in [32]. The concrete mixtures also included silica fume, which was incorporated by partially substituting cement with silica fume in varying amounts ranging from 5% to 20%. The test results indicated a significant reduction in both the strength and modulus values as the rubber content increased.

In [33], it was determined that in the regions where the environmental conditions are not harsh, use of concrete produced with 10% rubber aggregate is appropriate as it is economical and an effective way of recycling the discarded tires.

The addition of 5% waste tire rubber powder to the concrete mixture significantly enhanced its resistance to sulfate corrosion [34]. This improvement in durability indicates that the rubber powder helps to mitigate the deleterious effects of sulfate ions, which can cause severe deterioration in concrete structures over time. By incorporating waste tire rubber powder, the concrete becomes more resilient to sulfate attack, potentially extending the lifespan of structures exposed to sulfate-rich environments such as seawater, soil, and industrial effluents. This finding highlights the dual benefits of using waste tire rubber powder: it not only promotes sustainable recycling practices but also enhances the performance of concrete in challenging environmental conditions.

Paper [35] presents an experimental investigation comparing the performance of rubberized concrete to control mixed concrete in terms of chloride penetration depth, resistance to acid attack, and macrocell corrosion. Waste tire rubber, in the form of crumb rubber, replaced natural fine aggregates at varying levels from 0% to 20% in increments of 2.5%. Rubberized concrete demonstrated high resistance to aggressive environments, suggesting its suitability for areas prone to acid attack.

Implementing such a solution during the design phase offers several advantages. Firstly, it allows for careful planning and integration of damping properties into the structure’s design from the outset. This proactive approach ensures that damping elements are seamlessly incorporated into the construction process, minimizing the need for costly retrofits or modifications later on. Furthermore, addressing damping requirements during the design phase enables engineers to optimize the use of materials and resources, potentially reducing overall project expenses. By selecting appropriate materials with inherent damping properties or integrating damping mechanisms into structural components, designers can achieve effective vibration reduction without relying heavily on expensive external solutions. In contrast, attempting to implement damping measures during the operational phase can pose significant challenges and expenses. Retrofitting existing structures with damping devices or modifying construction methods to enhance damping capabilities often involves complex and disruptive procedures. Moreover, retrofitting may require additional labor, materials, and downtime, leading to increased project costs and potential operational disruptions. Therefore, by prioritizing damping considerations during the design phase, project stakeholders can effectively manage costs, streamline construction processes, and ensure optimal performance and longevity of the structure.

## 5. Conclusions

The conducted research program and the obtained results allow for the formulation of the following conclusions:

- Adding rubber granulate to the concrete mixture intended for palisades significantly enhances its vibration damping capabilities. This effect is especially important in large city centers where vibrations are particularly bothersome. Implementing such a solution can increase residents’ quality of life and reduce the impact of noise and vibrations on building infrastructure. The concrete mixture designed for palisades represents a balance between its vibration damping abilities and mechanical load-bearing strength. This is crucial because palisades must be sufficiently robust to ensure structural stability while reducing the impact of vibrations on the surroundings. The optimal rubber additive amount ensuring acceptable strength while maintaining optimal damping properties is 10%.
- Through the appropriate composition of chemical admixtures and mineral additives, it is possible to achieve a concrete mixture with high fluidity for up to 4 h from the first contact between cement and water. This is important for work in high-temperature conditions or during long-distance concrete transport, where maintaining fluidity for an extended period is crucial for the effectiveness of the construction process.
- The addition of rubber granulate practically does not change the air content in the concrete mixture or the depth of water penetration under pressure. This means that introducing rubber into the mixture does not worsen significant technical parameters of the concrete.
- The addition of rubber granulate proportionally reduces the compressive strength of concrete. The average compressive strength of concrete with 10% rubber granulate after 90 days is 35.6 N/mm^2^, compared to 45.8 N/mm^2^ for the reference concrete without rubber. This indicates that the rubber-modified concrete still meets the required strength criteria while providing enhanced vibration damping.

The research findings demonstrate that incorporating rubber granulate into concrete mixtures for palisades offers several benefits, particularly in enhancing vibration damping without significantly compromising other key properties. While the addition of rubber reduces compressive strength, a balance can be achieved by optimizing the amount of rubber to 10%, maintaining acceptable strength and damping properties. This innovation is especially useful in urban construction, where reducing vibrations can greatly improve the quality of life and structural integrity. Moreover, the high fluidity of the mixture, maintained for up to 4 h, ensures efficient workability in challenging conditions. Overall, these insights provide valuable guidance for the design and execution of construction projects involving concrete palisades.

## 6. Patents

The Polish Patent Office acknowledges that on 25 October 2021 an electronic application was received for the grant of a patent for the invention: Concrete Mixture with Increased Vibration Isolation. The application is marked with the following number: P.439299.

## Figures and Tables

**Table 1:** The recipe for the base concrete mixture.

		Origin	Quantity, kg/m^3^
Binders:	CEM III/A 42.5 N	Nowiny	360
	Fly ash	Łaziska	80
Water:		Water supply	162
Chemical additives:	EnviroMix 178	Chryso	3.15
Water-to-cement ratio		0.45	
The density of the concrete mixture, kg/m^3^:		2420	
Sand point, %:		40.8	
Amount of cement paste, dm^3^/m^3^:		341	
Amount of mortar, dm^3^/m^3^:		605	
Content of fine fractions ≤ 0.125 mm, kg/m^3^		497	

**Table 2:** The consistency of concrete mixtures with rubber granulate additives along with air content.

Additive	Dosage [%]	Slump Cone [mm]		Air Content [%]
		After 10 min	After 60 min	
Reference concrete C35/45	-	225	240	0.4
Rubber granulate 2/6 mm	10	235	250	0.6
	15	230	240	0.9
	20	210	230	1.2
	25	220	250	1.1
	30	205	230	1.0

**Table 3:** The results of the compressive strength tests of concrete after 7, 28, 56, and 90 days of curing—industrial test results.

Additive	Dosage [%]	Average Compressive Strength after Days [N/mm^2^]				Concrete Class
		7	28	56	90	
Reference concrete C35/45	-	26.2	55.4	58.0	61.2	C45/55
Rubber granulate 2/6 mm	10	23.1	42.9	45.0	50.9	C35/45
	15	18.2	36.6	38.9	42.9	C30/37
	20	16.4	33.7	36.7	40.4	C25/30
	25	15.5	28.4	30.1	33.8	C20/25
	30	13.8	25.3	28.8	30.1	C20/25

**Table 4:** The results of the water penetration depth test for concretes with rubber granulate additives.

Additive	Dosage [%]	Maximum Water Penetration Depth under Pressure According to PN-EN 12390-8 [mm] [17]
Reference concrete C35/45	-	8
Rubber granulate 2/6 mm	10	6
	15	11
	20	12
	25	15
	30	10

**Table 5:** The results of the examination of the C30/37 concrete mixture and hardened concrete with a 10% addition of rubber granulate were evaluated during the field trial.

Property	Property		
Consistency of concrete mixture—slump cone [mm] (according to PN-EN 12350-2) [25]	after 10 min 225	after 60 min 255	Consistency class S5
Air content [% vol.] (according to PN-EN 12350-7) [22]	after 10 min 0.7	after 10 min 0.5	-
Compressive strength [N/mm^2^]	after 7 days 25.4	after 28 days 47.8	Clas acc. PN-EN 206 C30/37 [26]
Maximum depth of water penetration under pressure [mm] (according to PN-EN 12390-8) [17] after the equivalent time according to PN-B-06265:2018-10—for CEM III—90 days [23]	8		

**Table 6:** The consistency behavior of the concrete mixture with a 10% addition of rubber granulates after dosing 0.8% by mass of cement of the CHRYSO Plast CER 50 admixture.

Mixture Type	Consistency—Cone Slump after Time [Minutes]						
	10	60	90	120	180	240	300
C30/37 XA1 S4/S5 D_max_ 16 Cl 0.20	225	250	260	250	245	235	220

## Data Availability

The original contributions presented in the study are included in the article, further inquiries can be directed to the corresponding author.
